# Association of complicated Baker’s cysts with knee pathologies as compared to simple Baker’s cysts

**DOI:** 10.1097/MD.0000000000038407

**Published:** 2024-06-07

**Authors:** Jeong Min Kim, Seok Kang, Joon Shik Yoon

**Affiliations:** a Department of Rehabilitation Medicine, Seoul National University Hospital, Jongno-gu, Seoul, Republic of Korea; b Department of Physical Medicine and Rehabilitation, Korea University Guro Hospital, Korea University College of Medicine, Guro-gu, Seoul, Republic of Korea.

**Keywords:** Baker’s cyst, MRI, popliteal cyst

## Abstract

Baker’s cysts (BCs) are known to be associated with intra-articular pathologies. BCs can be classified into 2 types: simple and complicated. Although some studies have focused on BC using magnetic resonance imaging (MRI), which is the gold standard examination, no study has compared knee MRI features in patients with simple and complicated BCs. To assess the relationship between the type of BC (simple vs complicated) and other knee pathologies using MRI. Seventy patients who underwent knee MRI examination due to symptomatic knee were retrospectively recruited from April 2011 to April 2021 at a single hospital. In the knee MRI images, the following were assessed: type (simple or complicated), morphology, volume of BCs, thickness of the suprapatellar recess, presence of synovial proliferation of the suprapatellar recess, grade of knee joint effusion, presence of meniscal tear, and extent of meniscal extrusion. The patients were classified into 2 groups according to the type of BC: simple BC and complicated BC. The differences between the 2 groups were evaluated for all variables. Finally, 52 patients were included in this study, 15 were classified as “simple BC” group and 37 as “complicated BC” group. The volume of complicated BC (median: 4.6, interquartile range – IQR: 1.6–12.4) was significantly greater than that of simple BC (median: 0.7, IQR: 0.3–3.7; *P* = .007). The presence of synovial proliferation in the suprapatellar recess was significantly higher in complicated BC (91.9%) than that in simple BC (46.7%; *P* = .001). The thickness of the suprapatellar recess was significantly greater in complicated BC (median: 7.5, IQR: 5.8–10.7) than that in simple BC (median: 4.3, IQR: 2.3–7.6; *P* = .020). The medial meniscus extrusion was greater in complicated BC (median: 4.1, IQR: 2.8–5.1) than that in simple BC (median: 2.5, IQR: 1.8–4.4; *P* = .037). After adjusting these *P*-values using the Holm method, only the presence of synovial proliferation in the suprapatellar recess remained significant (*P* = .010). Using knee MRI images, we demonstrated that complicated BCs are more associated with intra-articular pathologies than simple BCs; such as cyst volume, amount of the knee joint effusion, synovial proliferation and medial meniscal extrusion. Among them, the presence of synovial proliferation was the most significant factor associated with complicated BCs.

## 1. Introduction

Baker’s cyst (BC), or popliteal cyst, is a bursa consists of knee joint effusion which is bulged between the gastrocnemius and semimembranosus tendons.^[1,2]^ Most of the BCs are secondary BCs connected with knee joint space.^[1–6]^ BCs sometimes cause clinical symptoms such as pain or swelling. When the size become large, BC can cause knee stiffness or decrease flexibility.^[7,8]^ At times, it can be ruptured causing inflammation at surrounding structures. Also BC can compress adjacent popliteal vessels occurring thrombosis, or compress nerves causing neuropathy.^[2,9]^ Symptomatic patients with BCs can be treated by aspiration, corticosteroid injection surgical interventions.^[8,10–13]^

In adults, BCs are associated with other intra-articular pathologies, such as meniscal tear (usually medial meniscus), tear of cartilage or anterior cruciate ligament.^[13–16]^ In addition, BCs are related with knee osteoarthritis or rheumatoid arthritis.^[7,10,17]^ Therefore, it is necessary to diagnose not only BC but also other associated knee pathologies.

For noninvasively diagnosing BCs, ultrasound or magnetic resonance imaging (MRI) can be utilized.^[2,18–20]^ Although BC can be easily detected by ultrasound, visualizing associated other knee lesions by ultrasound is less sufficient compared to MRI. Also, ultrasound images are somewhat examiner-dependent. However, using MRI, clinicians can evaluate other intra-articular pathologies in detail more objectively, so it is gold standard for diagnosis.^[2,17,20,21]^

In some studies, BCs were classified by its appearance; simple BC and complicated BC. The simple BC is detected as anechoic, well-delineated cyst with thin synovial wall. The complicated BC is nonhomogenous, septated cyst with synovial thickening.^[5,10,22]^ Köroğlu et al^[10]^ demonstrated that relapse rate after cyst aspiration and steroid injection was significantly higher in complicated BC group compared to simple BC group. Park et al^[22]^ compared clinical, radiographic and ultrasound characteristics of simple BC and complicated BC. They suggested that complicated BC accompanies synovial proliferation and larger effusion in the suprapatellar recess more frequently compared to simple BC.

Although there were some studies focused on BC using MRI, to the best of our knowledge, there was no study comparing the knee MRI features in patients with simple BC and complicated BC. Therefore, the aim of this study is to assess the relationship between the type of BC (simple vs complicated) and other knee pathologies by using MRI, which is the gold standard examination.

## 2. Methods

### 2.1. Participants

The subjects were retrospectively recruited from April 2011 to April 2021 in Korea University Guro Hospital. The subjects were those who visited Korean University Guro Hospital due to symptoms such as knee pain, discomfort, and limited ROM and underwent MRI. Among them, only patients with BC on MRI were included. Patients who met following criteria were excluded: younger than 40 years, infectious arthritis, rheumatoid arthritis, crystal-associated arthritis, acute knee trauma, history of knee surgery, and in case of insufficient demographic data or images for research. Demographic data, such as age, sex, height and weight were obtained by reviewing medical records. Approval was made by the Korea University Guro Hospital Institutional Review Board (IRB No. 2021GR0102) and informed consent was waived by the institutional review board.

### 2.2. Collection of data from images

The images of knee plain radiographs and MRI, with patient information blinded, were analyzed. The collected data are described as follows.

#### 2.2.1. Kellgren–Lawrence (KL) grade measurement

The Kellgren–Lawrence (KL) grade was assessed in the knee plain radiograph images. The criteria of KL grade are as follows^[23,24]^:

1. Grade 1: doubtful narrowing of the joint space with possible osteophyte formation.
2. Grade 2: possible narrowing of the joint space with definite osteophyte formation.
3. Grade 3: definite narrowing of joint space, moderate osteophyte formation, some sclerosis, and possible deformity of bony ends.
4. Grade 4: large osteophyte formation, severe narrowing of the joint space with marked sclerosis, and definite deformity of bone ends.

#### 2.2.2. Knee MRI analysis

MRI was performed with the patients in supine position and their knee extended. 3.0 T MRI imaging unit scanners were utilized. T2-weighted or proton-density-weighted fat-suppressed images were mainly assessed. Sagittal, axial, and coronal views were analyzed.

To distinguish BC from other mass lesions, we identified the beak like cyst extension between the medial head of the gastrocnemius and the semimembranosus tendon.

The following contents were assessed by the knee MRI images; the type (simple BC or complicated BC), morphology (beak, crescent, and X-shaped), and volume of BC, thickness of the suprapatellar recess, presence of synovial proliferation of the suprapatellar recess, grade of knee joint effusion, presence of meniscal tear (medial and lateral), and extent of meniscal extrusion (medial and lateral) were assessed.

**Figure 1.**
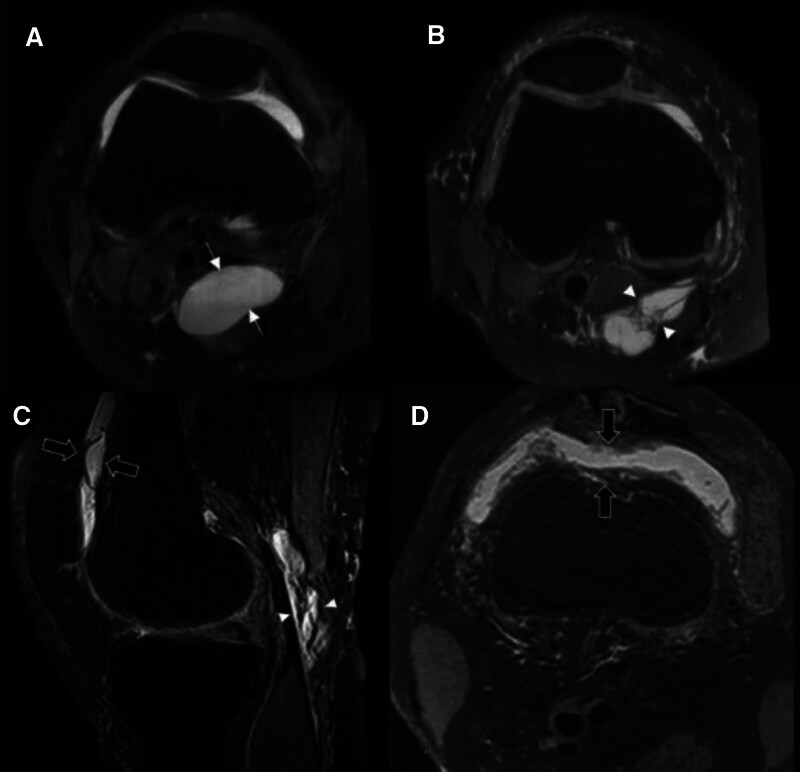
Axial and sagittal view of fat-suppressed proton-density MRI of the BC. (A) Simple BC (arrows) is characterized as homogenous signal intensity lesion with smooth well-defined borders and thin synovial wall. (B, C) Complicated BC (arrowheads) shows internal nonhomogeneity, septation, synovial thickening, and intracystic nodules or debris. (C, D) Synovial proliferation in the suprapatellar recess (black arrows) is observed in the knee with complicated BC. BC = Baker’s cyst, MRI = magnetic resonance imaging.

**Figure 2.**
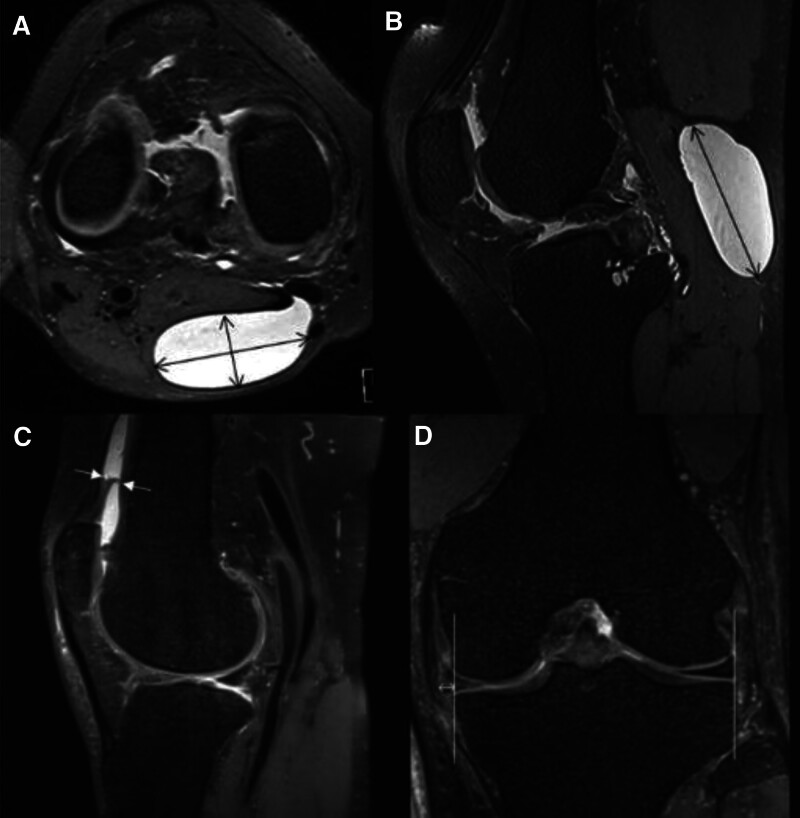
(A, B) Axial and sagittal views were used for examining the volume of BC (*V* = length × height × width × 0.52). (C), Sagittal view was used for measuring the thickness of the suprapatellar recess. (D) Coronal section with the largest volume of the medial tibial spine was used for assessing the medial and lateral meniscal extrusion. The vertical lines crossing the outer border of medial and lateral tibial plateaus were used as references to assess the extrusion of the meniscus. Only medial meniscal extrusion is seen in this image. BC = Baker’s cyst.

(1) The type of BC: The type of BC was classified into 2 categories – “simple BC” and “complicated BC.” The simple BC was characterized by homogenous signal intensity and well-defined borders with thin synovial wall. The complicated BC was defined as heterogeneous intracystic fluid, septated, irregular synovial thickening, and intracystic nodules or debris.^[5,10,22]^ The MRI samples of simple and complicated BCs are suggested in Figure 1.
(2) The morphology of BC: The morphology of BC was classified into 3 categories – crescent, beak, and X-shaped cysts.^[22,25]^
(3) The volume of BC: The volume of ellipsoids can be calculated with the “*V* = D1 × D2 × D3 × 0.52” formula (D1 = transverse diameter, D2 = anteroposterior diameter, D3 = longitudinal diameter). We applied this formula for estimating the volume of BC.^[10,22]^ The diameters are measured in the sagittal and axial views (Fig. 2).
(4) Thickness of the suprapatellar recess: In the sagittal image, the maximal anteroposterior diameter of the suprapatellar recess was assessed. It was measured as perpendicular to the cortex of femur (Fig. 2).
(5) Synovial proliferation of the knee joint: As suggested in Figure 1, the presence of synovial proliferation was assessed in the knee joint space including suprapatellar recess. Synovial proliferation can be detected as intermediate signal intensity, while the joint effusion as high signal intensity on T2-weighted images and lower signal intensity on T1-weighted images.^[26,27]^
(6) The grade of knee joint effusion: Among the Magnetic resonance imaging Osteoarthritis Knee Score (MOAKS), “effusion-synovitis” criteria were applied. It classifies joint effusion grade using knee MRI into 4 categories: grade 0 to 3^[28]^:
  o Grade 0 (none): physiologic amount;
  o Grade 1 (small): fluid continuous in the retropatellar space;
  o Grade 2 (medium): with slight convexity of the suprapatellar bursa;
  o Grade 3 (large): evidence of capsular distention.
(7) Presence of meniscal tear: The presence of medial and lateral meniscus tear was assessed, respectively.
(8) Extent of meniscal extrusion: As demonstrated in Figure 2, coronal images were used for measuring the extent of medial and lateral meniscal extrusion. The image where the medial tibial spine with its great volume was considered as a reference section. The extent of the medial meniscus extrusion was measured from the outer edge of medial tibial plateau. The extent of the lateral meniscal extrusion was measured from the outer edge of lateral tibial plateau.^[29]^

### 2.3. Data processing

The subjects were classified into 2 groups by the type of BC: simple BC versus complicated BC groups. Differences between the 2 groups were evaluated for all variables.

### 2.4. Statistical analysis

All analyses were conducted using IBM SPSS statistics version 26 and SAS version 9.4. *P*-values <.05 were considered statistically significant. Shapiro–Wilk test was used for testing the normality of data. Continuous variables with normal distribution were suggested as mean ± standard deviation (SD). Continuous variables not normally distributed were suggested as median and interquartile range (IQR). Categorical variables were suggested as frequency and percentage. Independent *t* test was used for evaluating the difference of normally distributed continuous variables between the 2 groups. Mann–Whitney *U* test was applied for evaluating the difference of not normally distributed continuous variables or ordinal variables between the 2 groups. For categorical variables, the Fisher exact test was used. *P*-values were adjusted using the Holm method.

## 3. Results

Among 70 patients, 18 were excluded by the exclusion criteria. Finally, 52 subjects were included in this study. There were 15 knees in “simple BC” group and 37 knees in “complicated BC” group. The demographic data of the subjects are described in Table 1.

**Table 1:** General characteristics of patients.

Parameter	Total (n = 52)		*P*-value
	Simple BC (n = 15)	Complicated BC (n = 37)	
Sex			.10
Male (n, %)	4 (26.7%)	9 (24.3%)	
Female (n, %)	11 (73.3%)	28 (75.7%)	
Age, yr (mean, SD)	63.3 ± 9.9	65.2 ± 9.2	.539
Height, cm (mean, SD)	159.1 ± 9.3	159.1 ± 7.1	.997
Weight, kg (median, IQR)	58.7 (54.0–69.0)	65.3 (56.0–74.0)	.110
Body mass index, kg/m^2^ (mean, SD)	24.4 ± 2.5	26.1 ± 3.4	.095

Statistical significance was set at *P* < .05. An independent *t* test was used to evaluate the differences in normally distributed continuous variables between the 2 groups. The Mann–Whitney *U* test was used to evaluate the difference in nonnormally distributed continuous variables between the 2 groups. Fisher’s exact test was used to analyze categorical variables.

BC = Baker’s cyst, IQR = interquartile range, SD = standard deviation.

The volume of complicated BC (median: 4.6, IQR: 1.6–12.4) was significantly greater than that of simple BC (median: 0.7, IQR: 0.3–3.7; *P* = .007). The presence of synovial proliferation in suprapatellar recess was significantly higher in complicated BC (91.9%) than in simple BC (46.7%; *P* = .001). The thickness of suprapatellar recess were significantly greater in complicated BC (median: 7.5, IQR: 5.8–10.7) than in simple BC (median: 4.3, IQR: 2.3–7.6; *P* = .020). Also, the medial meniscus extrusion was greater in complicated BC (median: 4.1, IQR: 2.8–5.1) than in simple BC (median: 2.5, IQR: 1.8–4.4; *P* = .037). After adjusting these *P*-values using the Holm method, only the presence of synovial proliferation in the suprapatellar recess was found to be significantly associated (*P* = .010; Table 2).

**Table 2:** Magnetic resonance imaging and radiographic findings of simple and complicated Baker’s cysts.

Parameter	Total (n = 52)		*P*-value	Adjusted *P*-value†
	Simple BC (n = 15)	Complicated BC (n = 37)		
Cyst morphology			.087	
Beak (n, %)	6 (40.0%)	7 (18.9%)		.522
Crescent (n, %)	6 (40.0%)	11 (29.7%)		
X-shaped (n, %)	3 (20.0%)	19 (51.4%)		
Cyst volume, mL (median, IQR)	0.7 (0.3–3.7)	4.6 (1.6–12.4)	.007*	.063
Synovial proliferation of the knee joint				
Yes (n, %)	7 (46.7%)	34 (91.9%)	.001*	.010*
No (n, %)	8 (53.3%)	3 (8.1%)		
Thickness of the suprapatellar recess, mm (median, IQR)	4.3 (2.3–7.6)	7.5 (5.8–10.7)	.020*	.160
Joint effusion grade				
0 (n, %)	1 (6.7%)	4 (10.8%)	.271	1.000
1 (n, %)	7 (46.7%)	7 (18.9%)		
2 (n, %)	5 (33.3%)	18 (48.6%)		
3 (n, %)	2 (13.3%)	8 (21.6%)		
Medial meniscal tear				
Yes (n, %)	3 (20.0%)	4 (10.8%)	.397	1.000
No (n, %)	12 (80.0%)	33 (89.2%)		
Lateral meniscal tear				
Yes (n, %)	14 (93.3%)	27 (73.0%)	.145	.725
No (n, %)	1 (6.7%)	10 (27.0%)		
Medial meniscal extrusion, mm (median, IQR)	2.5 (1.8–4.4)	4.1 (2.8–5.1)	.037*	.259
Lateral meniscal extrusion, mm (median, IQR)	0.0 (0.0–0.0)	0.0 (0.0–0.0)	.382	1.000
Kellgren–Lawrence grade				
I (n, %)	3 (20.0%)	8 (21.6%)	.374	1.000
II (n, %)	7 (46.7%)	9 (24.3%)		
III (n, %)	5 (33.3%)	16 (43.2%)		
IV (n, %)	0 (0.0%)	4 (10.8%)		

Statistical significance was set at *P* < .05.

BC = Baker’s cyst; IQR = interquartile range.

* The Mann–Whitney *U* test was used to evaluate the difference in nonnormally distributed continuous variables between the 2 groups. Fisher’s exact test was used to analyze categorical variables.

† *P*-values were adjusted by using Holm method.

## 4. Discussion

This study has demonstrated that the complicated BCs are more associated with intra-articular pathologies, such as larger cyst volume, larger amount of effusion in the suprapatellar recess, synovial proliferation and medial meniscal extrusion. Among them, the presence of synovial proliferation was the most significant factor associated with complicated BCs.

Consistent with the fact that most secondary BCs are connected to the knee joint space,^[1–5]^ some previous studies have suggested that BCs are associated with lesions within the knee joint.^[6,13–16]^ Balik et al retrospectively examined 45 knee MRIs of symptomatic patients. They demonstrated that increased volume of BC is significantly related with degeneration of cartilage, medial plicae, and increased knee joint effusion.^[6]^ Meniscal tear was found in 71% to 84% in knee with BCs.^[14–16]^ Stone et al^[16]^ evaluated 238 knee MRIs who had BCs. Seventy-four percent of them had meniscal tear, including complete or degenerative tear. Sansone et al^[14]^ examined 46 knee MRIs with BCs and found 94% of them had on, or more, disorders, such as meniscal tear (83%), chondral lesion (43%), and anterior cruciate ligament tears. Consistently, there was 1 or more pathologic findings in knee joint related with osteoarthritis in subjects in our study, who had BCs.

Only a few studies compared the characteristics of simple BC and complicated BC. The simple BC is characterized by an anechoic, well-delineated cyst with thin synovial wall. The complicated BC is nonhomogenous, septated cyst with synovial thickening.^[5,10,22]^ Köroğlu et al^[10]^ demonstrated that relapse rate after cyst aspiration and steroid injection was significantly higher in complicated BC group compared to simple BC group. Park et al^[22]^ also compared the characteristics of simple BC and complicated BC. They compared clinical, radiographic and ultrasound characteristics and demonstrated that complicated BC more frequently accompanies synovial proliferation and larger effusion in the suprapatellar recess compared to simple BC. However, there was no significant difference in osteophytes grade, effusion grade, meniscal tear, cyst volume, morphology, power Doppler findings, or KL grade. Compared to their results, in our study, it was consistent that synovial proliferation and larger effusion in the suprapatellar recess were significantly more associated with complicated BC than simple BC. Among them, the presence of synovial proliferation remained significant even after adjusting for *P*-values. Also, the presence of meniscal tear and the cyst morphology and KL grade were not significantly related to simple or complicated BC. However, in contrast to their results, in our study, larger cyst volume was more significantly associated with complicated BC than simple BC, although the significance diminished after adjusting the *P*-values. Similarly, we found that the extent of medial meniscal extrusion, although not statistically significant after *P*-value adjustment, was more severe in knees with complicated BC compared to simple BC. According to Crema et al,^[29]^ meniscal extrusion is associated with knee osteoarthritis processes such as meniscal tears, knee malalignment, and cartilage damage. In our study, MRI was used, which is more accurate and less examiner-dependent than ultrasound, so more accurate data would have been obtained compared to the previous studies.

Although the etiology of BCs is not clear, meniscal tear is considered as significantly associated factor of development of BCs.^[14,16]^ Stone et al^[16]^ used MRI, which is a gold standard examination for observing meniscus, and suggested that meniscal tear was present in 71% of knees with BC. Only 1 study investigated the type of BCs (simple or complicated) and presence of meniscus tear, and there was no significant difference.^[22]^ Also, among 47 knees with BC, meniscus was detected only in 15 (32%) knees. Their results were because; they used ultrasound which has limitation for visualizing meniscus. However, in our study, it was found that there was no significant association between the presence of meniscal tear and whether the BC is simple or complicated type. This result may be due to the degree or type of meniscus tear was not separately classified. If the severity of the meniscal tear was classified to multi-grade, the association between the medial tear and BC type could be obtained more accurately.

There are some limitations in this study. As this was a retrospective study, the clinical data such as pain severity, associated symptoms, detailed physical examination and lab findings were not consistently recorded in all subjects. In addition, although we analyzed data collected for 10 years, the number of samples in the 2 groups was not sufficient to be normally distributed. Future study including large number of subjects recruited prospectively is needed. Also, if response to the treatment is added, it would be more helpful in determining the treatment direction of the BC.

## 5. Conclusions

This study, using knee MRI, demonstrated that the complicated BCs are more associated with intra-articular pathologies compared to the simple BCs; such as cyst volume, amount of the knee joint effusion, synovial proliferation, and medial meniscal extrusion. Among them, the presence of synovial proliferation was the most significant factor associated with complicated BCs. Through this study, it would be helpful to understand the characteristics the type of BCs and to determine the treatment direction of BCs.

## Author contributions

**Conceptualization:** Jeong Min Kim, Joon Shik Yoon.

**Data curation:** Jeong Min Kim.

**Funding acquisition:** Joon Shik Yoon.

**Investigation:** Jeong Min Kim.

**Methodology:** Jeong Min Kim, Joon Shik Yoon.

**Supervision:** Joon Shik Yoon.

**Visualization:** Jeong Min Kim.

**Writing – original draft:** Jeong Min Kim.

**Writing – review & editing:** Jeong Min Kim, Seok Kang, Joon Shik Yoon.
